# Functional identification of organic cation transporter 1 as an atenolol transporter sensitive to flavonoids

**DOI:** 10.1016/j.bbrep.2015.06.005

**Published:** 2015-06-24

**Authors:** Yoshihisa Mimura, Tomoya Yasujima, Kinya Ohta, Katsuhisa Inoue, Hiroaki Yuasa

**Affiliations:** a Department of Biopharmaceutics, Graduate School of Pharmaceutical Sciences, Nagoya City University, 3-1 Tanabe-dori, Mizuho-ku, Nagoya 467-8603, Japan; b Department of Biopharmaceutics, School of Pharmacy, Tokyo University of Pharmacy and Life Sciences, 1432-1 Horinouchi, Hachioji, Tokyo 192-0392, Japan

**Keywords:** OCT1, Atenolol, Absorption, Intestine, Apple juice, Food–drug interaction

## Abstract

Atenolol, a β1-adrenergic receptor blocker, is administered orally and its intestinal absorption has recently been indicated to be mediated by carrier protein and reduced markedly by ingestion of some fruit juices, such as apple and orange juices. This could be postulated to be a problem arising from the interaction of some components of fruit juices with atenolol at a transporter involved in its intestinal uptake, but the responsible transporter and its interacting components have not been identified yet. In an attempt to examine that possibility, we could successfully find that human organic cation transporter 1 (OCT1/SLC22A1), which is suggested to be expressed at the brush border membrane of enterocytes, is highly capable of transporting atenolol. In this attempt, OCT1 was stably expressed in Madin-Darby canine kidney II cells and the specific uptake of atenolol by the transporter was found to be saturable, conforming to the Michaelis-Menten kinetics with the maximum transport rate (*V*_max_) of 4.00 nmol/min/mg protein and the Michaelis constant (*K*_m_) of 3.08 mM. Furthermore, the OCT1-specific uptake was found to be inhibited by various flavonoids, including those contained in fruit juices that have been suggested to interfere with intestinal atenolol absorption. Particularly, phloretin and quercetin, which are major components of apple juice, were potent in inhibiting OCT1-mediated atenolol transport with the inhibition constants of 38.0 and 48.0 µM, respectively. It is also notable that the inhibition by these flavonoids was of the noncompetitive type. These results indicate that OCT1 is an atenolol transporter that may be involved in intestinal atenolol uptake and sensitive to fruit juices, although its physiological and clinical relevance remains to be further examined.

## Highlights

• We explored an atenolol transporter from among the cation or anion transporters.
• OCT1 expressed on apical side in enterocytes has transport activity of atenolol.
• Transport of atenolol by OCT1 is inhibited by flavonoids.
• Phloretin and quercetin noncompetitively inhibit OCT1-mediated atenolol transport.

## 1 Introduction

Atenolol is a selective β1-adrenergic receptor blocker that is orally effective in the treatment of hypertension, angina pectoris, and cardiac arrhythmias [1], [2], [3]. This drug, of which the absorption is rather moderate with the bioavailability of about 50% [4], has generally been assumed to be absorbed by simple diffusion in the intestine [5]. However, based on recent findings of significant reductions in its absorption by simultaneous administration with apple juice [6] and with orange juice [7], it may be needed to assume that carrier-mediated uptake transport, which could be inhibited by some constituents of those fruit juices, is involved in its absorption. It may be possible that the disposition of this hydrophilic drug, which little undergoes metabolism and is eliminated almost exclusively (90%) by excretion into the urine in unchanged form after absorption [8], is regulated by membrane transporters.

Organic anion transporting polypeptide 1A2 (OATP1A2/SLCO1A2) and OATP2B1/SLCO2B1, which have recently been suggested to be involved in the intestinal uptake of various anionic drugs [9], can also be inhibited by such plant components, and it could lead to decreases in the absorption of their substrate drugs [10], [11]. It should be of interest to note that some β1-adrenergic receptor blockers structurally analogous to atenolol have been reported to be transported by OATP1A2 and OATP2B1 [12], [13], even though this class of drugs is cationic. Particularly, talinolol is known as an OATP1A2 substrate, and its absorption is also inhibited by flavonoids [12]. Thus, OATP1A2 and OATP2B1 may also contribute to the absorption of atenolol, but another possibility could not be excluded that some other transporters for cationic compounds may participate in that process. We, here, describe our successful attempt to identify human OCT1/SLC22A1 as an atenolol transporter, which may be involved in intestinal atenolol absorption. OCT1 is known to be typically expressed in the basolateral membrane of hepatocytes and contribute to extrusion of various cationic compounds including endogenous compounds and drugs, such as tetraethylammonium (TEA), 1-methyl-4-phenylpyrimidine (MPP^+^), acetylcholine and metformin [14], [15], [16]. Diphenhydramine, atropine and desipramine are, on the other hand, known as its typical inhibitors that are not transported. Recently, its presence and potential role in drug transport has also been suggested in the intestine in the brush border and basolateral membranes.

## 2 Materials and methods

### 2.1 Materials

[^14^C]Tetraethylammonium (TEA, 55 mCi/mmol) and [^3^H]1-methyl-4-phenylpyridinium (MPP^+^, 85 Ci/mmol) were obtained from American Radiolabeled Chemicals (St. Louis, MO, USA), [^3^H]atenolol (3.3 Ci/mmol) was from Moravek Biochemicals (Mercury Lane, Brea, CA, USA), and [^3^H]estrone-3-sulfate (54 Ci/mmol) was from PerkinElmer (Waltham, MA, USA). Unlabeled atenolol and Dulbecco’s modified Eagle’s medium (DMEM) were from Wako Pure Chemical Industries (Osaka, Japan). Fetal bovine serum (FBS) was obtained from Invitrogen (Carlsbad, CA, USA). All other regents were of analytical grade and commercially obtained.

### 2.2 Cell culture

Madin-Darby canine kidney II (MDCKII) cells and human embryonic kidney 293 (HEK293) cells were maintained at 37 °C and 5% CO_2_ in DMEM supplemented with 10% FBS, 100 U/ml penicillin, and 100 µg/ml streptomycin.

### 2.3 Plasmids

The plasmids carrying the cDNAs of OCT1/SLC22A1, OCT2/SLC22A2, OCT3/SLC22A3, OCTN1/SLC22A4 and OCTN2/SLC22A5 of human were those prepared in our previous study [17]. The plasmids carrying the cDNAs of OATP1A2 and OATP2B1 of human were prepared by using the cDNAs isolated by RT-PCR cloning from the human small intestine total RNA (Clontech, Mountain View, CA, USA) in the present study. In brief, an RT reaction was performed to obtain the cDNA mixture, using 1 µg of the total RNA, an oligo(dT) primer and ReverTra Ace (Toyobo, Osaka, Japan) as a reverse transcriptase. The cDNA of OATP1A2 was amplified by PCR, using KOD plus polymerase (Toyobo) and the following primers: forward primer, 5’-CCA GAT TTT AAG ACC AAC GC-3’ and reverse primer, 5’-TTC AAA GTT CCC CAG TGT AA-3’. These primers were designed on the basis of the sequence in GenBank (accession number NM_021094.3). PCR was performed using the following conditions: predenature at 94°C for 2 min and 33 cycles of (1) denature at 98°C for 10 s, (2) annealing at 52°C for 20 s, and (3) extension at 68°C for 90 s. The second PCR was performed using the PCR product as a template and a forward primer containing XhoI restriction site (underlined), 5′-GAA CTC G AGC AAC ATG GGA GAA ACT G-3′ and a reverse primer containing XbaI restriction site (underlined), 5′-GCT CTA GAG TTG TAC AGC ATG TTC TC-3′. The amplified cDNA product was incorporated into pCI-neo vector (Promega, Madison, WI, USA) at XhoI and XbaI sites, and the sequence of the final product was determined with an automated sequencer. The cDNA of OATP2B1 (GenBank accession number NM_007256. (4) was isolated similarly by RT-PCR cloning. The primers for PCR were as follows: forward primer, 5′-TGC TTC CTC TCC CCT GCT AAG-3′ and reverse primer, 5′-GAA GGT GAT CCA GGC GAG TG-3′. The second PCR was performed using the PCR product as a template and a forward primer containing XhoI restriction site (underlined), 5′-GAA CTC GAG GTC ATG GGA CCC AGG ATA G-3′ and a reverse primer containing XbaI restriction site (underlined), 5′- GTG TCT AGA GGA GGT ACT GCT GTG GC-3′. The amplified cDNA product was incorporated into pCI-neo vector at XhoI and XbaI sites and the sequence of the final product was determined with an automated sequencer.

### 2.4 Preparation of MDCKII cells stably expressing OCT1

MDCKII cells were transfected with the plasmid carrying the cDNA of OCT1 by using Lipofectamine 2000 (Invitrogen) as a transfection reagent, according to the manufacturer’s instructions, and cultured in DMEM supplemented with 10% FBS and 400 µg/ml G418 for 2 or 3 weeks. Antibiotic resistant clones were selected and tested for the transport of [^3^H]MPP^+^ as a probe substrate.

### 2.5 Uptake study in HEK293 cells transiently expressing OCTs, OATPs and OCTNs

HEK293 cells (2×10^5^ cells/ml, 1 ml/well) were grown on 24-well plates coated with poly-l-lysine for 12 h, transfected with the plasmid carrying the cDNA of each transporter by using Lipofectamine 2000, and cultured for 36 h for transient expression. The transient expression of each transporter was confirmed by observing the specifically induced uptake of its typical substrate in HEK293 cells transfected with the plasmid for the transporter. The substrates were [^3^H]TEA for OCT1,OCT2, OCT3, OCTN1 and OCTN2, and [^3^H]estrone-3-sulfate for OATP1A2 and OATP2B1. The cells in each well were preincubated in 1 ml of substrate-free uptake buffer, that is Hanks’ solution (136.7 mM NaCl, 5.36 mM KCl, 0.925 mM CaCl_2_, 0.812 mM MgSO_4_, 0.441 mM KH_2_PO_4_, 0.385 mM Na_2_HPO_4_, and 25 mM d-glucose) supplemented with 10 mM HEPES (pH 7.4), for 5 min and uptake assays were started by replacing the substrate-free uptake buffer for preincubation with uptake buffer containing [^3^H]atenolol (0.25 ml). All the procedures were conducted at 37 °C. After uptake for 3 min, assays were stopped by addition of ice-cold substrate-free uptake buffer (2 ml) and the cells were washed two times with 2 ml of the same buffer. The cells were solubilized in 0.5 ml of 0.2 M NaOH solution containing 0.5% sodium dodecyl sulfate at room temperature for 1 h and the associated radioactivity was measured by liquid scintillation counting, using 3 ml of Clear-sol I (Nacalai Tesque, Kyoto, Japan) as a scintillation fluid, for the evaluation of uptake. Cellular protein content was determined by the BCA method (BCA Protein Assay Reagent Kit; Thermo Fisher Scientific, Waltham, MA, USA), using bovine serum albumin as the standard. Uptake assays were also conducted in mock cells, which were transfected with empty pCI-neo vector, to estimate nonspecific uptake.

### 2.6 Uptake study in MDCKII cells stably expressing OCT1

MDCKII cells stably expressing OCT1 (1.5×10^5^ cells/ml, 1 ml/well) were grown on 24-well plates for 48 h to confluence. The assays of [^3^H]atenolol uptake, which was for 3 min in regular experiments, were conducted by the same procedures as those described for assays in HEK293 cells transiently expressing OCT1. [^3^H]MPP^+^ was used as a substrate in some experiments to assess the OCT1-mediated transport characteristics of this typical substrate for comparison with those of [^3^H]atenolol. To examine the effect of pH, Hanks’ solution was supplemented with 10 mM MES, instead of HEPES, and adjusted to pH 5.5. To examine the effect of flavonoids and some other inhibitors, test compounds were added only to the uptake solution containing [^3^H]atenolol so that they were present in the solution only during uptake period. In experiments to examine the effect of BaCl_2_ as an agent to depolarize the plasma membrane, a chloride salt-based buffer (145 mM NaCl, 3 mM KCl, 1 mM CaCl_2_, 0.5 mM MgCl_2_, 5 mM HEPES, and 5 mM d-glucose) was used to avoid precipitation of Ba salts. Uptake assays were also conducted in mock cells, which were transfected with empty pCI-neo vector, to estimate nonspecific uptake. The specific uptake of atenolol or MPP^+^ by OCT1 was estimated by subtracting its uptake in mock cells from that in transporter-transfected cells.

The expression of OCT1 was confirmed by a greater uptake of [^3^H]MPP^+^ (5 nM) in the cells expressing OCT1 (170.1±11.1 fmol/mg protein, *n*=4) than in mock cells (12.9±0.3 fmol/mg protein, *n*=4), where the uptake was evaluated for 3 min at 37 °C and pH 7.4 and the experiments were duplicated in each of 2 preparations of cells, giving 4 as a total number.

### 2.7 Data analysis

The saturable transport of atenolol by OCT1 was analyzed by assuming Michaelis-Menten type carrier-mediated transport represented by the following equation: *v*=*V*_max_×*s*/(*K*_m_+*s*). The apparent parameters of the maximum transport rate (*V*_max_) and the Michaelis constant (*K*_m_) were estimated by fitting this equation to the experimental profile of the uptake rate (*v*) *versus* the concentration (*s*) of atenolol as the substrate, using a nonlinear least-squares regression analysis program, WinNonlin (Pharsight, Mountain View, CA), and the *v*^-2^ as the weight. The value of *v* was estimated as the mean of duplicate determinations in a preparation of cells. The parameters are presented as the means±S.E. of those estimated in multiple preparations of cells.

Based on the assumption of noncompetitive inhibition, *V*_max_ in the presence of an inhibitor (*V*_max,i_) can be related to that in its absence (*V*_max,0_), using the inhibitor concentration (*i*) and the inhibition constant (*K*_i_), as follows: *V*_max,i_=*V*_max,0_/(1+*i*/*K*_i_). Based on this equation, the *K*_i_ was estimated using the mean values of *V*_max,i_ and *V*_max,0,_ and the experimentally designated value of *i*.

When *s* is much smaller than *K*_m_ (*s* « *K*_m_), the *v* in the presence of noncompetitive inhibitor can be described as follows: *v*=*v*_0_/(1+(*i*/*IC*_50_) *^n^*). The half maximal inhibitory concentration (*IC*_50_) was estimated together with the Hill coefficient (*n*) by fitting this equation to the experimental profile of *v versus i*, with *v* in the absence of inhibitors (*v*_0_) fixed at the observed value.

Experimental data are presented as the means±S.E. Statistical analysis was performed by using Student's *t*-test or, when multiple comparisons were needed, analysis of variance followed by Dunnett's test, with *p*<0.05 considered significant.

## 3 Results

### 3.1 Identification of OCT1 as a transporter capable of transporting atenolol

**Fig. 1:**
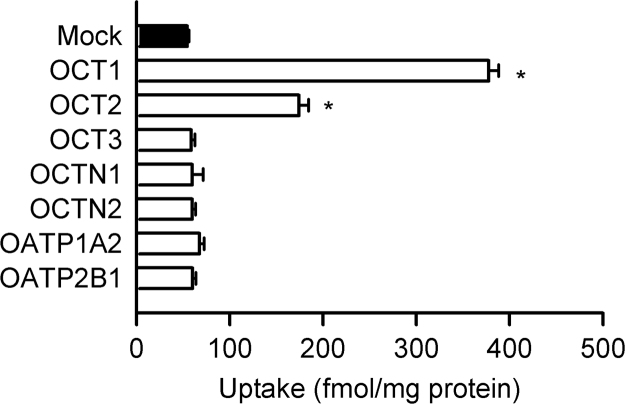
Screening of candidate transporters for atenolol in HEK293 cells transiently expressing each of them. Data are presented as means±S.E. (*n*=3), where the experiments were triplicated in a preparation of cells. The uptake of [^3^H]atenolol (100 nM) was evaluated for 3 min at 37°C and pH 7.4. *, *p*<0.05 compared with uptake in mock cells.

To identify transporters that are capable of transporting atenolol, we had several candidate transporters, which are reportedly expressed in the small intestine [18], [19], expressed transiently in HEK293 cells and examined the cellular uptake of atenolol. As shown in Fig. 1, the uptake of atenolol was, compared with that in mock cells, increased most extensively in the cells expressing OCT1 and, to a lesser extent, in those expressing OCT2, indicating the ability of these transporters to transport atenolol, but not in those expressing the other transporters. Because OCT1 has been suggested to be present in the brush border membrane of enterocytes [20], while OCT2 has been suggested to be present in the basolateral membrane [18], we assumed that OCT1 could be a transporter that may be involved in the intestinal uptake of atenolol and conducted further characterization of its function for atenolol transport.

### 3.2 Functional characterization of OCT1 as an atenolol transporter

**Fig. 2:**
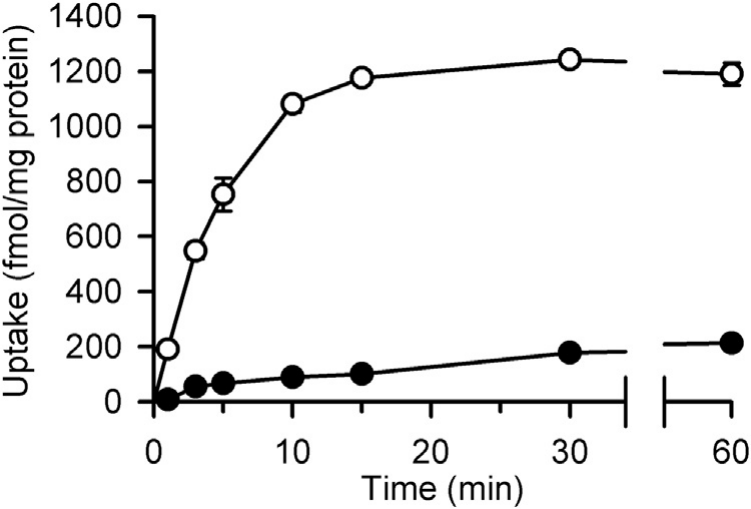
Time course of the uptake of atenolol in MDCKII cells stably expressing OCT1. Data are presented as means±S.E. (*n*=4), where the experiments were duplicated in each of 2 preparations of cells, giving 4 as a total number. The uptake of [^3^H]atenolol (100 nM) was evaluated at 37°C and pH 7.4 in cells expressing OCT1 (open circle) and in mock cells (closed circle).

For more detailed characterization of atenolol transport by OCT1, we prepared MDCKII cells stably expressing OCT1 and first examined the time course of the uptake of atenolol. As shown in Fig. 2, the uptake of atenolol in the cells expressing OCT1 was much greater than that in mock cells, consistent with the suggestion in HEK293 cells transiently expressing OCT1 that atenolol is a good substrate of this transporter, and increased in proportion to time up to 3 min. Based on this time course, we set the uptake period to be 3 min in subsequent experiments to evaluate atenolol transport across the plasma membrane within the initial uptake phase.

**Fig. 3:**
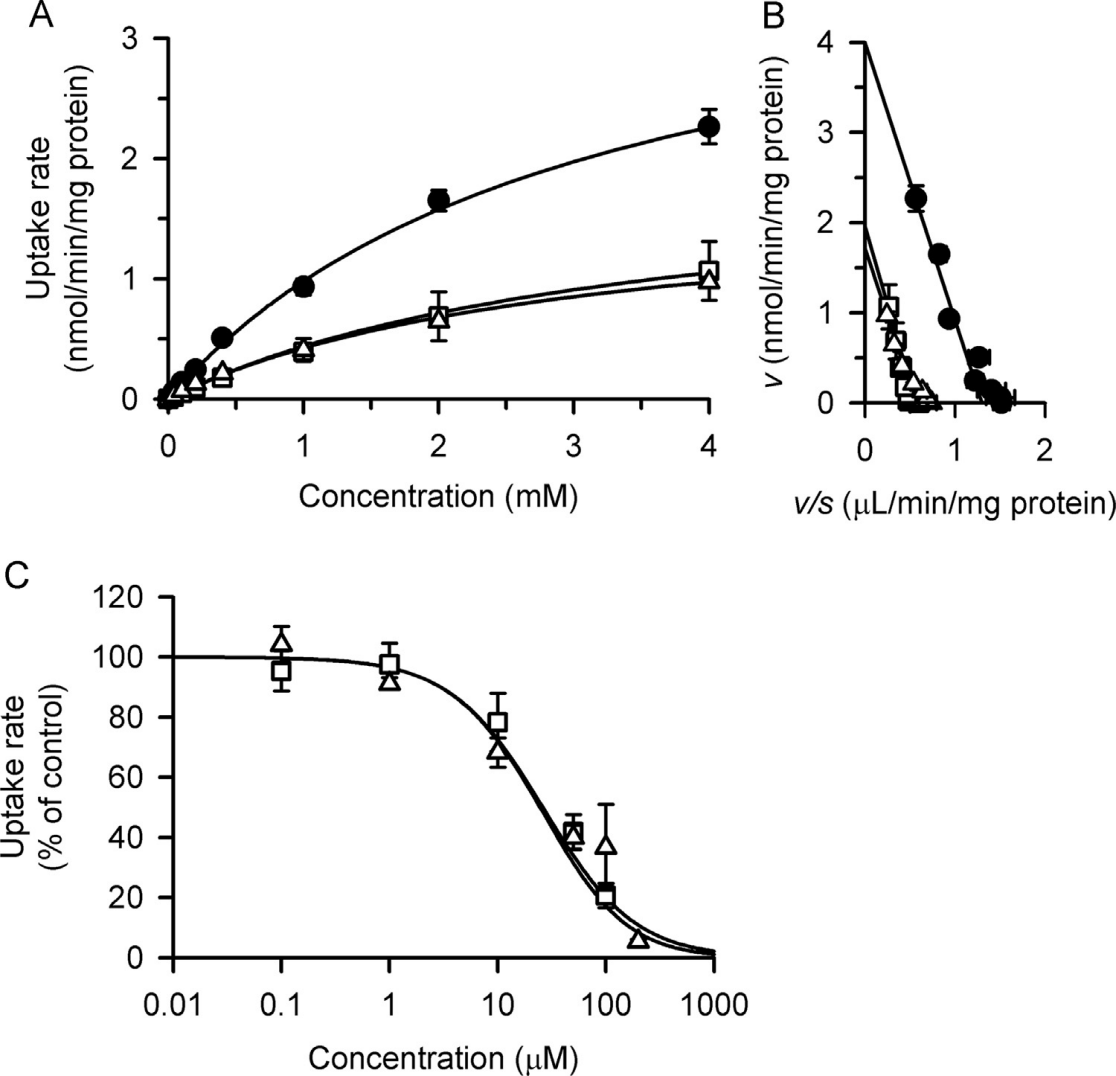
Kinetic analysis of atenolol transport by OCT1 stably expressed in MDCKII cells. (A) The rate of specific atenolol uptake by OCT1 was evaluated for 3 min at 37 °C and pH 7.4 at varied concentrations of atenolol in the absence of flavonoids (closed circles) or in the presence of phloretin (open triangles, 50 µM) or quercetin (open squares, 50 µM). Solid lines show profiles based on the respective sets of the mean values of *V*_max_ and *K*_m_. The values of *V*_max_ (nmol/min/mg protein) and *K*_m_ (mM) are 4.00±0.22 and 3.08±0.17, respectively, for control, 1.71±0.12 and 3.04±0.26, respectively, in the presence of phloretin, and 1.96±0.55 and 3.46±0.78, respectively, in the presence of quercetin. (B) Eadie-Hofstee plots for OCT1-specific atenolol uptake. (C) The rate of OCT1-specific uptake of [^3^H]atenolol (100 nM) was evaluated for 3 min at 37 °C and pH 7.4 in the presence of varied concentrations of phloretin (open triangles) and quercetin (open squares). The values of *v*_0_ are 192 and 193 fmol/min/mg protein, respectively, in experiments using phloretin and quercetin. The values of *IC*_50_ (µM) and *n* are 28.4±4.7 and 1.1±0.1, respectively, for phloretin, and 35.3±9.1 and 1.3±0.2, respectively, for quercetin. The uptake rate was estimated as the mean of duplicate determinations in a preparation of cells. The data of uptake rate and parameters are presented as means±S.E. of those estimated in 3 preparations of cells (*n*=3).

The concentration-dependent profile of the rate of specific atenolol uptake by OCT1 was found to be highly saturable and conformed to the Michaelis-Menten kinetics with a *K*_m_ of 3.08 mM and a *V*_max_ of 4.00 nmol/min/mg protein (Fig. 3A). The specific uptake rate of atenolol by OCT1 was, when assessed at 100 nM as a low concentration below the *K*_m_ and within the linear phase of the Michaelis-Menten kinetics, sensitive to pH, being significantly reduced to 65.7±4.5 from 193.2±3.9 fmol/min/mg protein (means±S.E., *n*=4, *p*<0.05) when pH was lowered to 5.5 from 7.4, and was also sensitive to the membrane potential, being significantly reduced to 149.4±6.6 from 321.5±4.9 fmol/min/mg protein (means±S.E., *n*=4, *p*<0.05) by depolarization of the plasma membrane by the addition of BaCl_2_ in the medium. Since atenolol is supposed to be almost completely ionized in the range of pH 5.5 to pH 7.4, based on its reported pKa of 9.4 [21], the status of its ionization was unlikely to be a factor to affect its transport in the present study. These characteristics of atenolol transport were consistent with the functional characteristics reported for OCT1 [14] and also with those observed in the present study for the specific uptake of MPP^+^ as its typical substrate. The specific uptake rate of MPP^+^ (5 nM), which was evaluated for 3 min, was reduced to 21.5±1.0 from 56.6±3.7 fmol/min/mg protein (means±S.E., *n*=4, *p*<0.05) when pH was lowered to 5.5 from 7.4, and was reduced to 42.6±0.9 from 98.1±1.5 fmol/min/mg protein (means±S.E., *n*=4, *p*<0.05) by depolarization of the plasma membrane by BaCl_2_. The experiments to assess the effects of pH and membrane depolarization were duplicated in each of 2 preparations of cells, giving 4 as a total number.

### 3.3 Effect of phloretin and quercetin on atenolol uptake by OCT1 stably expressed in MDCKII cells

We then examined the effect of phloretin and quercetin, which are the major flavonoids contained in apple juice [22], on OCT1-specific atenolol uptake in MDCKII cells stably expressing the transporter, because the absorption of atenolol is reportedly reduced most extensively by apple juice [6]. As shown in Fig. 3A, both phloretin and quercetin inhibited OCT1-specific atenolol uptake potently by reducing *V*_max_ without altering *K*_m_ at their concentration of 50 µM. Therefore, as shown more clearly by Eadie-Hofstee plots in Fig. 3B, both phloretin and quercetin were suggested to inhibit OCT1 noncompetitively, and the *K*_i_ values of these flavonoids were estimated to be 38.0 and 48.0 µM respectively. These values were comparable with the *IC*_50_ values of 28.4±4.7 and 35.3±9.1 µM for the respective flavonoids, confirming their noncompetitive inhibitory actions on OCT1 (Fig. 3C).

A recent study by Mandery et al. indicated no influence of quercetin on MPP^+^ transport by OCT1 [23]. However, the OCT1-specific uptake rate of MPP^+^ (5 nM) was also found to be inhibited by quercetin in the present study, being reduced to 34.2±0.5 fmol/min/mg protein in the presence of quercetin from 58.5±6.1 fmol/min/mg protein in its absence (means±S.E., *n*=4, *p*<0.05), where the experiments were duplicated in each of 2 preparations of cells, giving 4 as a total number. It is also notable that quercetin was found to inhibit the OCT1-specific transport of DAPI, a fluorescent probe substrate, in our previous study [24]. Therefore, although the reason why Mandery et al. could not observe the influence of quercetin on MPP^+^ transport by OCT1 is unknown, our findings strongly suggest that OCT1 is most likely to be sensitive to quercetin.

### 3.4 Effect of various compounds on atenolol transport by OCT1 stably expressed in MDCKII cells

**Fig. 4:**
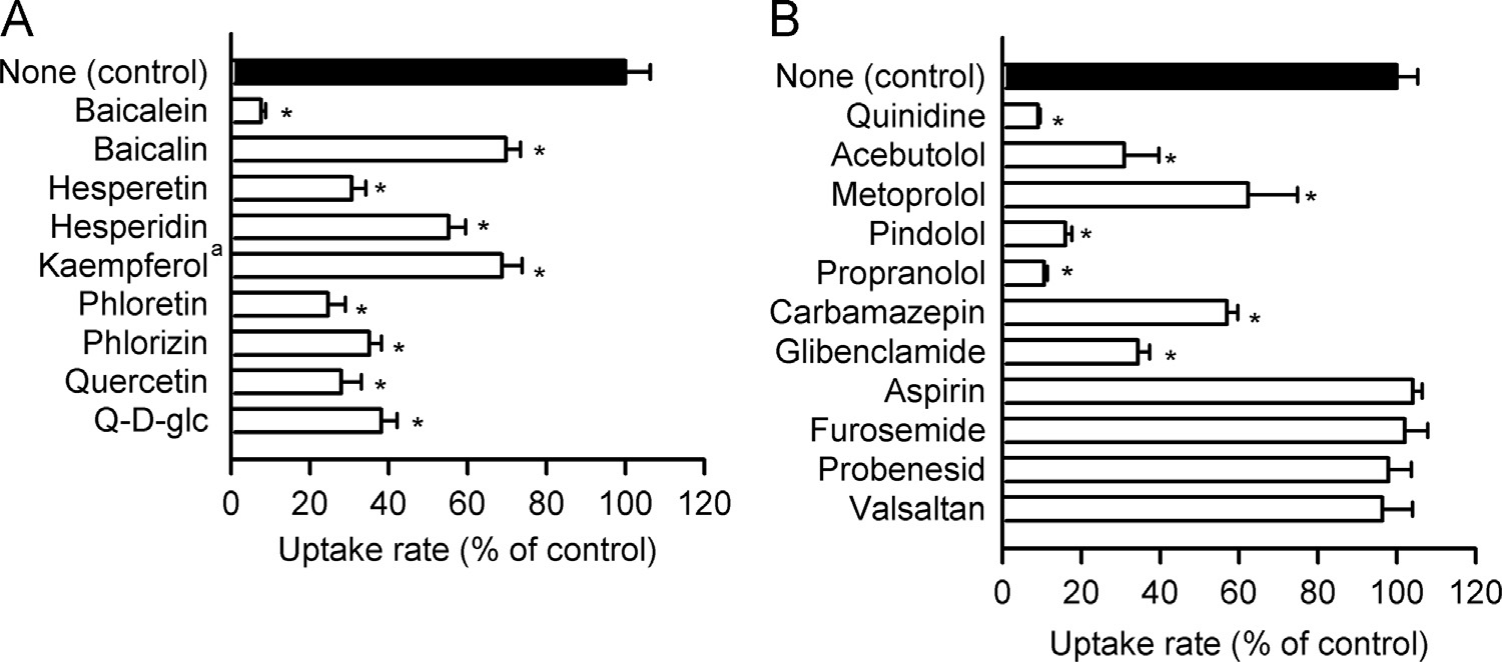
Effect of various compounds on the uptake of atenolol by OCT1 stably expressed in MDCKII cells. (A) The specific uptake of [^3^H]atenolol (100 nM) was evaluated for 3 min at 37 °C and pH 7.4 in the presence of a flavonoid (10 µM for kaempferol^a^ and 100 µM for the others) or in its absence. Q-d-glc, quercetin-3β-d-glucoside. The control value of specific uptake rate was 204 fmol/min/mg protein. (B) The specific uptake of [^3^H]atenolol (100 nM) was evaluated for 3 min at 37 °C and pH 7.4 in the presence of a drug (100 µM) or in its absence. The control value was 126 fmol/min/mg protein. Data are presented as means±S.E. (*n*=4), where the experiments were duplicated in each of 2 preparations of cells, giving 4 as a total number. *, *p*<0.05 compared with control.

To assess the function of OCT1 as an atenolol transporter in more detail, we here examined the effect of various compounds, including phloretin, quercetin, and several other flavonoids on OCT1-specific atenolol uptake in MDCKII cells stably expressing the transporter. Kaempferol, which is another major flavonoid contained in apple juice, was found to inhibit OCT1-specific atenolol uptake significantly at a rather low concentration of 10 µM (Fig. 4A), although its effect could not be examined at higher concentrations because of the limitation of its solubility. Hesperetin, which is a major flavonoid in orange juice, exhibited an inhibitory action comparable to those of phloretin and quercetin at its concentration of 100 µM. Phlorizin, quercetin-3ß-d-glucoside and hesperidin, which are the glucosides of phloretin, quercetin and hesperetin, respectively, also exhibited inhibitory activities comparable to those of their aglycones [22]. Baicalein, which is a flavonoid contained in *Scutellaria baicalensis* [25], exhibited the most potent inhibitory activity, although baicalin as its glycoside exhibited an only moderate inhibitory activity.

The specific uptake of atenolol was inhibited by quinidine (Fig. 4B), suggesting that atenolol is likely to be competed by this OCT1 substrate [14]. On the other hand, N-methyl nicotinamide (NMN), which is known not to inhibit OCT1 activity at this concentration [14], did not affect atenolol uptake by OCT1. OCT1-specific atenolol uptake was also found to be inhibited by several cationic drugs including ß1-adrenergic receptor blockers analogous to atenolol, but not by anionic drugs. These ß1-adrenergic receptor blockers may also be transported by OCT1.

## 4 Discussion

We here have described the novel function of OCT1 as an atenolol transporter. OCT1 is well known to be involved in the elimination of various organic cationic compounds in the liver [18]. Therefore, OCT1 may be also in operation for atenolol uptake in the liver. However, the role of this process in the elimination of atenolol is unclear, because atenolol is known to be almost exclusively excreted into the urine from the kidney [4], [8]. Recently, OCT1 is reported to be localized to the apical membrane in polarized Caco-2 cells, which are widely used as a model of the human small intestinal epithelial cell [20], [26]. However, the localization of OCT1 in the epithelial cells in the human tissue remains inconclusive, because it has also been suggested that OCT1 plays a role in exporting its substrates across the basolateral membrane in the intestine as well as liver and kidney [18], [27]. Taken all these together, OCT1 may contribute to the transport of cationic compounds at both the apical and basolateral membranes in enterocytes.

Fruit juices have been known to inhibit enzymes involved in metabolism and transporters involved in drug absorption in the intestine [28], [29], [30]. Some components of grapefruit juice have been shown to be potent inhibitors of cytochrome P450 (CYP) 3A4, one of the major drug metabolism enzymes [31]. Furthermore, OATPs, which could be involved in the absorption of various anionic drugs in the intestine, has also been suggested to be inhibited by constituents of fruit juices [10], [11], [12], [13]. It was previously reported that the plasma concentrations of atenolol are significantly decreased by co-administration of apple juice and orange juice [6], [7]. According to pharmacokinetic analysis, *C*_max_ and AUC were significantly reduced by 68% and 81%, respectively, by apple juice and by 49% and 40%, respectively, by orange juice. However, the half life of its elimination was not changed [6], [7]. This suggested that apple and orange juices only affected the absorption process, but not the elimination process. Our present study showed that OCT1-mediated atenolol transport can be inhibited by various flavonoids, including those in apple and orange juices. In addition, inhibition by phloretin and quercetin, which are the major components of apple juice [22], [32], were found to be noncompetitive. This would be a finding to support the suggestion that OCT1 participates in atenolol absorption in the small intestine. Although OATP2B1, which is also known to be inhibited by fruit juices, has been suggested to be involved in the absorption of some β1-adrenergic receptor blockers [13], its genetic variation is reported not to affect the disposition of atenolol [6] and, furthermore, we could not detect transport activity of OATP1A2 and OATP2B1 for atenolol (Fig. 1).

In conclusion, we have successfully identified OCT1 as an atenolol transporter. Atenolol absorption in the small intestine may be mediated by OCT1 rather than OATP1A2 and OATP2B1, which have been suggested to be involved in the absorption of some β1-adrenergic receptor blockers. The transport activity of OCT1 was found to be reduced by phloretin and quercetin, which are the major components of apple juice, and also by several other flavonoids. This could provide a possible molecular basis for the mechanism behind the inhibition of atenolol absorption by fruit juices, although the effect of those flavonoids and also some typical OCT1 inhibitors on atenolol absorption needs to be assessed in humans to clarify the role of OCT1 in atenolol absorption and the role of flavonoids in the inhibition of atenolol absorption by apple juice in the future.
